# From a Polymorphous Low-Grade Neuroepithelial Tumor to a Glioblastoma in an Adult Patient with FGFR3-TACC3 Fusion: A Case Report and Literature Review of the Molecular Profile

**DOI:** 10.3390/curroncol33030165

**Published:** 2026-03-13

**Authors:** Lorena Gurrieri, Nada Riva, Alessia Tomassini, Giulia Ghigi, Maurizio Naccarato, Patrizia Cenni, Daniela Bartolini, Chiara Cavatorta, Luigino Tosatto, Monia Dall’Agata, Laura Ridolfi

**Affiliations:** 1 Clinical and Experimental Oncology, Immunotherapy, Rare Cancers and Biological Resource Center, IRCCS Istituto Romagnolo per lo Studio dei Tumori (IRST) “Dino Amadori”, Via P. Maroncelli 40, 47014 Meldola, Italy; 2 Neurosurgery Unit, “Maurizio Bufalini” Hospital, 47521 Cesena, Italy; 3 Radiotherapy Unit, IRCCS Istituto Romagnolo per lo Studio dei Tumori (IRST) “Dino Amadori”, 47014 Meldola, Italy; 4 Neuroradiology Unit, “Maurizio Bufalini” Hospital, 47521 Cesena, Italy; 5 Radiology Unit, IRCCS Istituto Romagnolo per lo Studio dei Tumori (IRST) “Dino Amadori”, 47014 Meldola, Italy; 6 Pathology Unit, “Maurizio Bufalini” Hospital, 47521 Cesena, Italy; 7 Unit of Biostatistics and Clinical Trials, IRCCS Istituto Romagnolo per lo Studio dei Tumori (IRST) “Dino Amadori”, 47014 Meldola, Italy

**Keywords:** PLNTY, glioblastoma, seizure, fibroblast growth factor receptor, case report, WHO 2021

## Abstract

From an epidemiological perspective, polymorphous low-grade neuroepithelial tumor (PLNTY) represents a small proportion of brain tumors encountered in epilepsy surgery series. Their rarity and relatively recent recognition likely contribute to underdiagnosis and poor prognosis. In terms of histopathological features, they are similar to oligodendrogliomas. Molecular analyses can be used to show the fusion between fibroblast growth factor receptor (FGFR3) and transforming acidic coiled coil (TACC) proteins, which most commonly results in progression towards glioblastoma (GBM). We report a case of a 62-year-old man who underwent left frontal craniotomy to remove a frontal mass. Histologically, the glial lesion consisted of elements associated with oligodendroglia-like features. Immunohistochemistry was positive for glial fibrillary acidic protein (GFAP), oligodendrocyte transcription factor 2 (OLIG2), and α-thalassemia X-linked mental retardation syndrome (ATRX) nuclear expression, but negative for isocitrate dehydrogenase 1 (IDH1) and BRAF-V600E. Next-generation sequencing showed the *FGFR-TACC3* fusion, and taken together, these findings supported the final diagnosis of PLNTY. During follow-up, the patient underwent a second neurosurgery, where histological evaluation indicated a GMB. This article presents clinical and radiological data, morphology, immunohistochemistry, molecular features, and treatment to enhance the clinical and pathological understanding of PLNTY with *FGFR3-TACC3* fusion for all professionals involved in medical decisions.

## Simple Summary

Polymorphous low-grade neuroepithelial tumor (PLNTY) is a recently recognized low-grade epilepsy-associated neoplasm characterized by MAPK pathway alterations that frequently involve FGFR3, but malignant transformation has not been well documented. We report a rare adult case of PLNTY progressing to glioblastoma, highlighting the molecular evolution of the tumor. Histopathological evaluation and immunohistochemistry were performed at initial diagnosis and at recurrence, and targeted next-generation sequencing was used to assess genomic alterations, with particular focus on FGFR3 status. The primary lesion showed morphological and molecular features consistent with PLNTY, including FGFR3 alteration. At recurrence, the tumor demonstrated high-grade histological features and additional molecular changes consistent with glioblastoma, suggesting clonal evolution. This case underscores the importance of comprehensive molecular profiling in low-grade glioneuronal tumors. Further studies are needed to clarify the mechanisms driving malignant transformation and to determine whether FGFR3 alterations may represent potential therapeutic targets or biomarkers of progression.

## 1. Introduction

Polymorphous low-grade neuroepithelial tumor of the young (PLNTY), formerly regarded as a provisional entity, is recognized as a distinct tumor type in the 2021 World Health Organization (WHO) Classification of Tumors of the Central Nervous System (CNS) [1].

The designation “polymorphous low-grade neuroepithelial tumor of the young (PLNTY)” refers to a recently characterized, epilepsy-associated neoplasm initially delineated by Huse et al. In 2017 [2]. It typically affects children and young adults and is almost always defined by an oligodendroglioma-like morphology, widespread CD34 immunopositivity, and alterations involving the MAP kinase signaling cascade. Only a limited number of additional case reports have been published since then [3,4,5,6,7,8,9,10,11,12], mostly in younger individuals. Among these studies, Riva et al. (2018) describe the only case in a middle-aged patient [12].

The temporal lobe is the most common site of origin, and epileptic seizures are the predominant presenting manifestation [13]. PLNTY generally follows an indolent clinical course resembling that of WHO Grade I tumors, though an official histologic grade is yet to be defined. Complete surgical excision remains the treatment of choice, aiming both at mass reduction and seizure control, but recurrence appears to correlate with subtotal resections [14].

PLNTY pathogenesis involves activation of the mitogen-activated protein kinase (MAPK) pathway, most often through *BRAF V600E* mutations or *FGFR2/FGFR3* gene fusions (such as *FGFR3::TACC3*). These molecular aberrations are typically mutually exclusive and occur in over 90% of reported cases [15,16].

Herein, we describe a rare case of PLNTY with an *FGFR3–TACC3* fusion in a 62-year-old man, showing progression toward GBM.

## 2. Clinical Summary

**Figure 1:**
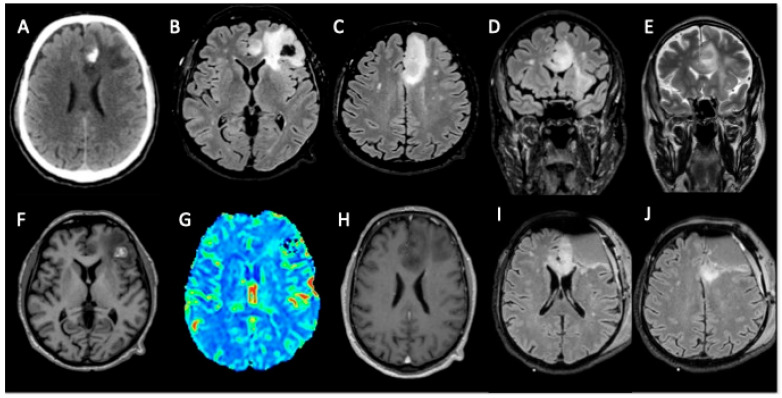
Polymorphous low-grade neuroepithelial tumor of the young (PLNTY) in a 62-year-old man with epileptic seizure scan: (**A**) MRI FLAIR (**B**–**D**,**I**,**J**), T1WI (**F**), T2WI (**E**), post-contrast T1-weighted images (**H**), DSC perfusion (**G**). A cortico-subcortical infiltrative tumor is present in the left frontal region, involving the corpus callosum through which it extends to the right cingulate gyrus. Perilesional edema, calcifications, and hemorrhagic components are evident. No enhancement is observed after contrast administration. The lesion does not show any significant increase in perfusion (rCBV). The postoperative MRI study (**I**,**J**) shows residual tumor in the genu of the corpus callosum and in the left frontomesial cortico-subcortical region.

A 62-year-old man was admitted to a primary hospital presenting with a tonic–clonic seizure, which caused a loss of consciousness and violent muscle contractions, and no other comorbidities were present in his medical history. A neuroradiological assessment was performed using a brain computed tomography (CT) scan, which revealed a subcortical intracerebral hemorrhage in his left frontal lobe. Brain magnetic resonance imaging (MRI) showed a cortical–subcortical tumor infiltration in his superior frontal gyrus, cingulate gyrus, and the anterior part of the corpus callosum, through which the tumor extended to the right cingulate gyrus, with no pathological enhancement or perfusion index increase. The patient underwent awake left frontal craniotomy to remove the mass, and his postoperative recovery showed no complications. Motor aphasia was addressed through speech therapy, and antiepileptic drugs were prescribed. A post-surgical brain MRI showed a residual tumor in the corpus callosum (Figure 1).

## 3. Pathological Findings

Histological features showed a moderately cellular glial neoplasm, consisting of elements with hyperchromic nuclei that are occasionally vesicular and enlarged or have evident nucleoli associated with oligodendroglial-like elements. Some dystrophic calcifications were found, and there were focal microcystic aspects of the fundus, which were also fibrillar, and perivascular lymphoid accumulations. Only sporadic mitoses were present (up to 1/10 HPF), but no obvious necrosis or vascular proliferation was observed. In addition, there were focal hypercellular areas in the absence, however, of mitotic increase.

**Figure 2:**
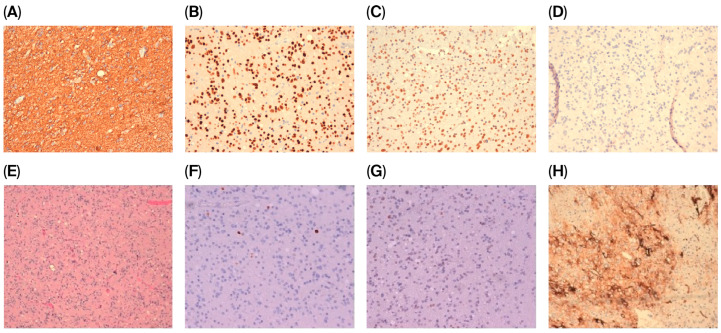
A moderately cellular neoplasm consisting of monomorphic cells with a rounded nucleus and clear cytoplasm (20× magnification); no necrosis or microvascular proliferation is present. (**A**) GFAP; (**B**) OLIG-2 reactivity; (**C**) ATRX positivity; (**D**) IDH-1 test; (**E**) hematoxylin; (**F**) Ki67 labeling index ≈ 2%; (**G**) weak expression of p53; (**H**) strong positive expression of CD34 in extravascular area.

Immunohistochemical profiling demonstrated reactivity for GFAP, OLIG2, and ATRX (Figure 2A–C). Staining for isocitrate dehydrogenase 1 (IDH1) (Figure 2D) and BRAF V600E was absent. The Ki-67 labeling index indicated a low proliferative activity (approximately 2%) (Figure 2F), and nuclear p53 expression was observed in fewer than 10% of tumor cells (Figure 2G). Diffuse and intense CD34 immunoreactivity was identified (Figure 2H), and fluorescence in situ hybridization (FISH) analysis did not reveal 1p/19q co-deletion. As per the National Rare Tumor Guidelines, the histopathology diagnosis was revised by another expert pathologist, agreeing that the combination of these features supported the diagnosis of a polymorphous low-grade neuroepithelial tumor (PLNTY). Integrative molecular NGS profiling showed a fusion between *FGFR3* (ex. 17) and *TACC3* (ex. 10). Because FGFR represents a target in gliomas and glioneuronal tumors, as reported in EANO guidelines, its immunohistochemical expression can be used for prescreening for GBM using a molecular test for *FGFR3::TACC3* fusion.

## 4. Clinical Outcome

**Figure 3:**
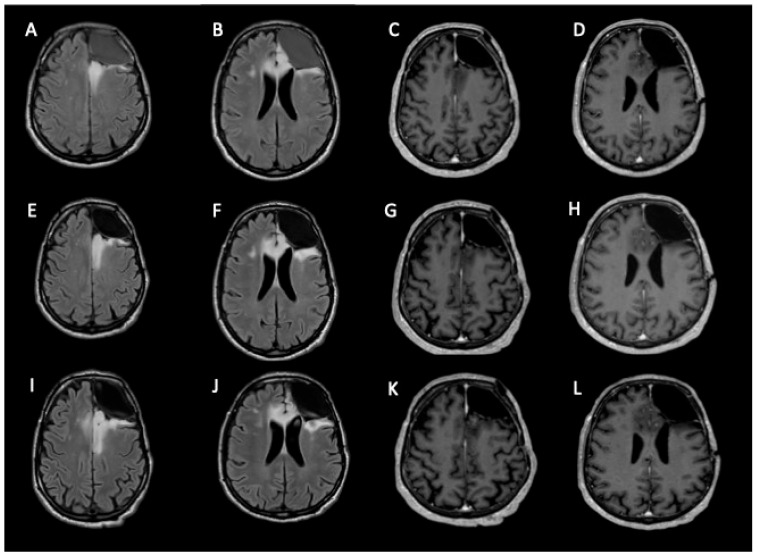
MRI follow-up studies were performed at 4, 10 and 16 months after surgery. The images shown are MRI FLAIR (**A**,**B**,**E**,**F**,**I**,**J**) and post-contrast T1-weighted images (**C**,**D**,**G**,**H**,**K**,**L**). There is progression and infiltration of the residual tumor through the genu of the corpus callosum, involving the left frontomesial cortico-subcortical region and ipsilateral cingulate gyrus, with extension to the contralateral frontomesial region and cingulate gyrus. Subtle areas of contrast enhancement are observed in the left frontomesial region and ipsilateral cingulate gyrus (**G**,**H**,**K**,**L**).

The Neuro-oncological Multidisciplinary Board of our institute discussed the case, opting for no adjuvant treatment, and the patient was placed in a 3-month follow-up program. From March 2022, the sequential brain MRI showed a slow, progressive increase in residual disease without seizures. We closely followed up until November 2022, at which point we noticed an increase in enhancement (Figure 3).

**Figure 4:**
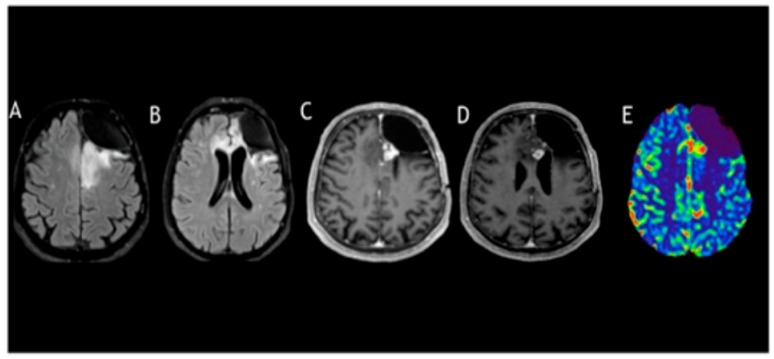
Follow-up MRI at 24 months after surgery and post-radiotherapy. The images shown are MRI FLAIR (**A**,**B**), post-contrast T1-weighted images (**C**,**D**), and DSC perfusion (**E**). Tumor progression is evident, with an increase in the infiltrative lesion (**A**,**B**) and areas of contrast enhancement (**C**,**D**) associated with elevated cerebral blood volume (**E**).

In January 2023, the patient completed his radiation therapy at a total dose of 54.4 gray. Unfortunately, after 4 months, an MRI showed disease progression again. From a neurological point of view, he had a partial seizure, and in July 2023, he underwent neurosurgery to remove the lesion, with contrast enhancement present in the preoperative MRI (Figure 4).

## 5. Pathological Findings

**Figure 5:**
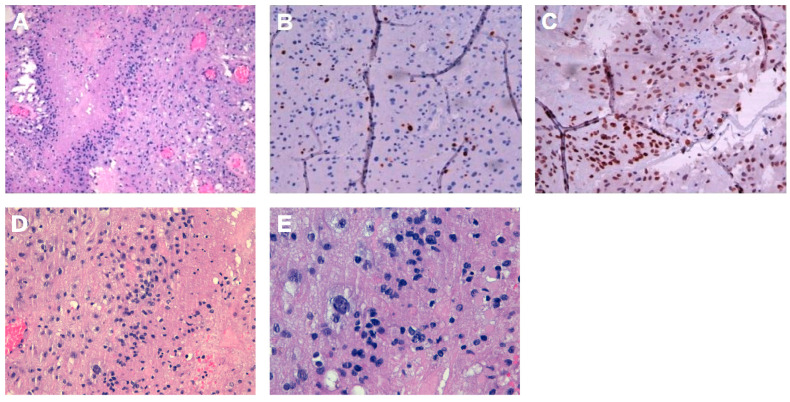
Cellular neoplasm quiescence is characterized by bulky, pleomorphic cells, interspersed with smaller monomorphic cells. Areas of pseudopalisading necrosis and microvascular proliferation are present: ((**A**) 10× magnification) hematoxylin; ((**B**) 20× magnification) Ki67 labeling index ≈ 8%; ((**C**), 20× magnification) intense diffuse expression of p53; ((**D**), 20× magnification) hematoxylin; ((**E**), 40× magnification) hematoxylin.

The histological features were as follows: a mild cellular glial neoplasm consists of medium-sized elements, which are monomorphic and have a hyperchromic nucleus, and larger-sized elements, with abundant cytoplasm, discrete nuclear pleomorphism, and sometimes evident nucleoli. Diffuse cystic aspects and some dystrophic calcifications were found. Some mitoses are present (up to 3/10 HPF), and foci of necrosis with pseudopalisading of the nuclei and areas of vascular proliferation were observed. During immunohistochemical investigations (Figure 5A), the neoplastic cells were positive for GFAP and Olig2 and negative for IDH1 and BRAF. ATRX expression is preserved (85%), and the proliferation rate, as quantified by Ki67, was 8% (Figure 5B), while p53 expression was 80% (Figure 5C). There is focal positivity for CD34, and the final diagnosis suggests the evolution of low-grade polymorphic neuroepithelial tumor (PLNTY) into IDH1-negative GBM (grade 4 according to CNS WHO V edition). Furthermore, MGMT methylation was 8%, and NGS profiling was negative for *FGFR3–TACC3* fusion.

## 6. Clinical Outcome

**Figure 6:**
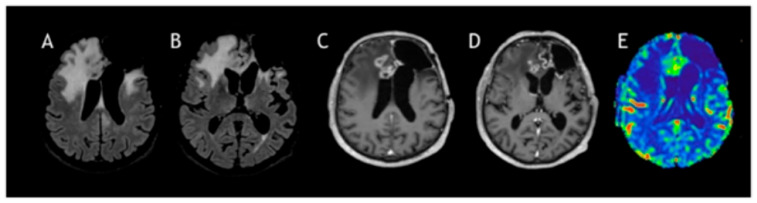
Progression of glioblastoma, using techniques MRI FLAIR (**A**,**B**), post-contrast T1-weighted images (**C**,**D**), and CBV map (**E**). Follow-up studies show tumor progression with increased infiltration of the corpus callosum and bilateral frontomesial regions, associated with mass effect, perilesional edema, irregular contrast enhancement, and elevated CBV.

Radiotherapy was not possible due to the previous irradiation doses and fields, and, as a result, the patient only started chemotherapy with oral administration of temozolomide at 150 mg/mq/day for five consecutive days for the first cycle; doses were increased to 200 mg/mq/day for 5 days every 28 days. Electroencephalography (EEG) post-surgery showed a left temporal slow wave, but there was no specific indication of a seizure. The patient continued with the regimen of AED levetiracetam 1500 BID and lacosamide 100 mg BID. After four cycles of MRI and PET FET, tumor progression was seen, and the patient started a second line with Regorafenib at full dose until seven cycles, after which clinical and radiological progression was monitored (Figure 6).

## 7. Discussion

Genetic rearrangements affecting *FGFR2* and *FGFR3* have been reported in PLNTY, with various fusion events being implicated. In the cohort described by Surrey et al. [17], several *FGFR2*-related fusions were detected, including a rare *FGFR2–CTNNA3* variant. In their seminal report, Huse et al. identified multiple gene fusions involving *FGFR2* and *FGFR3,* such as *FGFR3–TACC3, FGFR2–KIAA198*, and *FGFR2–CTNNA3* [2]. These rearrangements were found to be mutually exclusive with BRAF mutations. Clearly, all tumors demonstrated histologic features consistent with low-grade behavior, but no malignant transformation was reported during the follow-up.

Functionally, these *FGFR* fusion events promote ligand-independent receptor dimerization and subsequent autophosphorylation, leading to persistent activation of downstream components of the MAP kinase signaling cascade. Fusions involving *FGFR* and *TACC* genes have demonstrated oncogenic potential in GBM models and are identified in approximately 3% of GBM cases [18,19].

Our case presents an unusual and clinically significant progression from a presumed low-grade PLNTY to a GBM in an adult patient, with the *FGFR3-TACC3* fusion in the initial lesion being retained. This observation reveals important considerations regarding the malignant potential of PLNTY-like tumors and the role of *FGFR3-TACC3* as a possible driver of tumor progression. Golub et al. [20] described a similar phenomenon in which tumors initially diagnosed as PLNTY demonstrated features mimicking high-grade gliomas, and in some cases, displayed clear malignant transformation. Their findings highlighted that the presence of *FGFR3-TACC3* fusion is not exclusive to PLNTY and may appear in high-grade gliomas, including GBM, suggesting a spectrum of biological behavior potentially modulated by additional molecular events. Notably, Golub et al. emphasized the diagnostic challenge posed by tumors with overlapping histologic and molecular features between PLNTY and high-grade gliomas. They propose that FGFR3-TACC3 fusion, while a hallmark of PLNTY, is not pathognomonic and must be interpreted in the broader context of histopathology, age, and clinical course. In their case studies, no malignant progression was observed at 15 months of follow-up. Subsequent case series published by Ida et al., in a cohort of 13 patients, reported a predominance of temporal lobe involvement and recurrent *FGFR2* fusions [16]. Similarly, Chen et al. described three cases that were all treated via gross total resection, one of which harbored one *FGFR3-TACC3* and two *BRAF V600E* mutations [5], but despite these driver events, none of the patients demonstrated progression or transformation to GBM during follow-up. Among the cases summarized in Table 1, only Bale et al.’s study shows some resemblance to our patient, although it involved a 15-year-old patient with *FGFR3–TACC3*-positive PLNTY who developed radiological recurrence at 17 months, requiring repeat surgery and adjuvant treatment [21]. However, despite the more aggressive clinical course and management, a definitive diagnosis of GBM was not established. Broggi et al. focused on PLNTY developing in a middle-aged patient without malignant evolution [22], providing context that makes our case more relevant. Unlike previously reported cases, our patient was over 60 years old and exhibited a documented evolution from a tumor fulfilling the diagnostic criteria for PLNTY to a GBM, with acquisition of high-grade histological features and aggressive clinical behavior. A limitation of our case is the lack of assessment for *CDKN2A/2B* deletion, an alteration increasingly linked to high-grade progression in PLNTY. We focused on the presence of *FGFR3–TACC3* fusion in both phases, supporting a clonal relationship and suggesting true malignant progression rather than misclassification or sampling bias at initial diagnosis.

## 8. Conclusions

While the existing literature consistently presents PLNTY as a low-grade, indolent neoplasm with a favorable prognosis, our case is, to our knowledge, one of the first clear examples of transformation into GBM. Our findings highlight that, in adults, PLNTY with an *FGFR3-TACC3* fusion may not always follow an indolent clinical course. This fusion can lead to features more characteristic of GBM—both molecularly and morphologically—underscoring the importance of comprehensive molecular diagnostics to guide accurate classification, prognosis, and therapeutic planning.

## Figures and Tables

**Table 1:** Principal case report of PLNTY in the literature.

Study (Year)	No. of Cases	Age (Years)	Tumor Location	FGFR Alterations	BRAF V600E	Treatment	Progression/Evolution to GBM
Huse et al., 2017 [2]	10	4–32	Temporal (most common), frontal, occipital	FGFR3–TACC3 (1); FGFR2 fusions (2)	3 cases	Gross total resection (GTR)	No malignant transformation reported during follow-up
Bale et al., 2021 [21]	1	15	Left mesial temporal	FGFR3–TACC3	Negative	GTR → recurrence at 17 months → near-total resection + proton RT + temozolomide	Recurrence with higher-grade features; no definitive GBM diagnosis reported
Chen et al., 2020 [5]	3	Mean 15	Cortical (temporal predominance)	FGFR3–TACC3 (1)	2 cases	GTR	No malignant progression during follow-up
Ida et al., 2021 [16]	13	5–52 (median 16)	Predominantly temporal	FGFR2 fusions (multiple partners)	Present in subset	Surgical resection	No GBM transformation reported
Broggi et al., 2021 [22]	1	50	Left temporal	Not specified (MAPK pathway activation)	Not specified	GTR	No malignant evolution reported
Golub et al., 2023 [20]	1	59	Temporal lobe	FGFR3–TACC3 + TERT promoter mutation	Negative	GTR	No progression at 15-month follow-up

## Data Availability

The datasets generated and/or analyzed during the current study are available from the corresponding author upon reasonable request.
